# Exploring factors influencing number of fouls in soccer

**DOI:** 10.3389/fpsyg.2025.1510928

**Published:** 2025-05-20

**Authors:** Xuan Liu, Yuxin Zuo, Hamza Saghir Aslam, Seungmo Kim, Ming Fu

**Affiliations:** ^1^Department of Sports and Health Sciences, Hong Kong Baptist University, Kowloon Tong, Hong Kong SAR, China; ^2^Department of Health and Physical Education, The Education University of Hong Kong, Tai Po, Hong Kong SAR, China; ^3^Institute of Physical Education and Training, Capital University of Physical Education and Sports, Beijing, China

**Keywords:** soccer fouls, referees, distance, temperature, league stage

## Abstract

Fouls are common events in soccer games, and their occurrence is influenced by a number of factors. Based on data from the Amisco Pro^®^ semi-automated computer tracking system, this study explores factor influencing the numbers of fouls in soccer such as referees' running distance, foul decision distance, team strength difference, various temperature conditions, and league stages. A total of 480 soccer games from the 2018 and 2019 Chinese Super League were collected. Multiple linear regression and one-way ANOVA were employed for statistical analysis, with the significance level set at *p* < 0.05. The results showed that referees' running distance had a significant negative influence on the number of fouls, with *R*^2^ = 0.078. However, foul decision distance and the strength difference between teams were not significantly correlated with the number of fouls. Matches played in cool conditions (COOL, temperature < 20.0°C) had significantly more fouls than those played in hot (HOT, 25.1–30.0°C) and very hot (VHOT, >30.0°C) conditions. In contrast, no significant differences in the number of fouls were found between WARM (29.63 ± 6.72) and HOT (25.1–30.0°C) or VHOT (>30.0°C) with *p* > 0.05. In addition, Stage 1 had significantly fewer fouls than stage 2, with *p* < 0.05. On the other hand, no significant differences were found between Stage 1 and Stage 3 and between Stage 2 and Stage 3 (*p* > 0.05). This study highlights the impact of referees' active running on reducing the number of fouls in soccer, as well as the differences in number of fouls under various temperature conditions and stages. The findings provide insightful information for referees and the management department to anticipate the frequency of fouls and develop strategies to reduce fouls, thereby improving game quality. Future studies could explore the effects of additional environmental factors, such as stadium attendance and running track on the number of fouls.

## Introduction

Fouls are an integral part of soccer matches and occur frequently in matches (Gümüşdag et al., 2011). Since 1863, when The Football Association in England published the first Laws of the Game, officially defining football rule and establishing punishments for fouls (e.g., handball, violence), referees have served as the primary enforcers of these laws and the decision-makers for fouls committed by players (Curry, 2020). According to the Laws of the Game, fouls can be categorized in two types: those punishable by direct free kicks and those resulting in indirect free kicks (The International Football Association Board (IFAB), 2024). Number of fouls refers to the total fouls committed by both teams in a game (Yuan et al., 2024). Studies indicate that during the 2023–2024 season, the England Premier League (EPL) averaged 21.5 fouls per match while the German Bundesliga reported a slightly higher average of 21.9 fouls (Fournier, 2024). Fouls are often used tactically to disrupt opponent's counterattack or promising attacks (Englund, 2022; Lepschy et al., 2018). However, excessive fouls can lead to frequent match stoppages and even escalate into physical fights between teams (Joseph, 2015; Fields et al., 2009).

Previous studies have identified some factors influencing foul frequency. For example, Nevill et al. (2002) and Unkelbach and Memmert (2010) found that crowd noise significantly influenced referees' decisions, while Lex et al. (2015) demonstrated that players' vocalizations had no significant influence on foul calls. In addition, home advantage refers to the consistent finding that home teams win more often than away teams (Lago-Peñas and Lago-Ballesteros, 2011), and has been linked to foul distribution. For instance, Goumas (2012) reported that home teams received fewer fouls than away teams. The COVID-19 pandemic provided a unique opportunity to examine the influence of audiences on home advantage. Multiple studies reported that home advantage declined, with the number of fouls for home teams significantly increasing during matches played without spectators (Endrich and Gesche, 2020; Leitner and Richlan, 2021; Webb, 2021). In addition, Han et al. (2020) found that the number of fouls significantly declined after the application of the Video Assistant Referee system (VAR) in the Chinese Super League (CSL). Therefore, these findings highlight the need to further investigate the effects of air temperature and explore additional factors, including team strength disparities, game stages, and referees' running performance, on the number of fouls.

A soccer match is officiated by a group of referees, consisting of one Main Referee (hereafter referred to as “the referee”), two Assistant Referees (AR), and a fourth official [FO; (The International Football Association Board (IFAB), 2024)]. Moreover, Video Assistant Referees (VAR) and an Assistant VAR (AVAR) are used as additional support referees in some professional soccer matches such as the EPL, La Liga, the German Bundesliga, Serie A, the FIFA World Cup, and the Chinese Super League (CSL; Han et al., 2020). During a match, other referees (e.g., ARs, FO, VARs) have the right to provide information and advice to the referee on some foul situations, especially in relation to major or controversial fouls [e.g., penalties, yellow and red cards; (The International Football Association Board (IFAB), 2024)]. However, all fouls must be decided by the referee after running and observing the match (Kranjec et al., 2010). Consequently, referees, especially the referee, need to have an excellent level of physical fitness and their fitness test scores are important criteria for referees' promotion and qualification (Chiu, 2022). In this study, the running performance of the referee refers to the referees' total running distance throughout the game and the distance between the referee and the location of the foul when adjudicating it (Riiser et al., 2019). Referees are encouraged to run actively and minimize the distance from the referee to the foul incident location when making decisions (Mallo et al., 2010). Johansen and Erikstad (2021) found that decision accuracy is highest when referees' decision distance is within 10 m. With high decision accuracy, players may not feel angry about the judgment and are less likely to commit fouls as revenge. In addition, the active movement of the referee can create a sense among players that they are being closely monitored, potentially reducing the occurrence of unnecessary infringements. Referees are also expected to verbally warn players before a potential foul (Mascarenhas et al., 2006). Therefore, the referees' running performance (i.e., active ball running distance, decision-making distance) during a match may influence the occurrence of fouls. However, the relationship between referees' running performance (i.e., running distance and decision-making distance) and the number of fouls remains unclear.

Team quality is represented by the team's ranking at the end of the season, which has been commonly used in soccer tactical and technical analysis (Kong et al., 2022; Villaseca-Vicuña et al., 2024). The difference in team quality may be a significant factor affecting the number of fouls in soccer matches. Lago-Peñas (2012) demonstrated that soccer players' running performance can be influenced by the quality of the opposition. The contest for ball possession may become intense, resulting in more physical contact and a higher number of fouls when the two evenly matched teams compete. Previous studies have reported that the weaker team might commit more tactical fouls to restrict or hinder their opponents' attack (Liu et al., 2016). However, there is an extremely limited amount of research examining the impact of the difference in team quality on the number of fouls in soccer matches to date.

The number of fouls may also vary under different air temperatures during the game. Guy et al. (2015) found that running performance in elite middle- and long-distance athletes was negatively impacted at air temperatures above 25°C. The extent of performance decline correlates with both the duration of the event and the magnitude of temperature deviation from the optimal range of 10–15°C for endurance performance (Ely et al., 2007). In soccer matches, high-speed running, sprinting, tackling, accelerating, and decelerating occur intermittently, requiring a higher level of physiological and neuromuscular capacity (Harper et al., 2019; Stewart et al., 2019). Motion analysis data from the 2014 FIFA World Cup revealed a significant decline in high-intensity activities (e.g., sprinting) and high-intensity running distance at 34°C compared to 19°C (Nassis et al., 2015). Laboratory experiments have shown that increasing core and muscle temperatures result in approximately a 10% reduction in repeated-sprint performance (Drust et al., 2005). This impairment is consistent across both dry and humid heat conditions (Hayes et al., 2014). Kang et al. (2024) compared football players playing at temperatures under 21°C and over 29°C and reported that higher heart rate responses were found for players when playing at temperatures over 29°C, while more successful passes were observed for players when playing at temperatures under 21°C. Consequently, in high air temperature environments, the compromised physical ability may lead to fewer physical contacts and fouls. For example, Yuan et al. (2024) initially explored the effects of weather conditions on the number of fouls and found that air temperature and precipitation could influence the number of fouls. The study showed that there was a 7.6% decrease in players' running distance and a 23.1% decline in the number of fouls committed by CSL players in games played at temperatures over 30°C compared to games played at temperatures under 15°C. Although Yuan et al. (2024) study pioneered the research on the effects of temperature on the number of fouls, a significant limitation remains. All the data were collected from the CSL from 2015 to 2017, when there was no VAR. Therefore, it is crucial to explore the number of fouls across different temperatures under prevailing conditions (with VAR).

A regular season usually lasts more than 6 months, which could be equally divided into the early stage, middle stage, and the last stage (Zhou et al., 2019). The average number of fouls may vary across different stages. For example, in the early stage of the season, players are expected to be equipped with ample physical and psychological preparation after the off-season (Alexander, 2020). Players vigorously participate in body confrontation and challenges, which may lead to an increase in fouls. As the league progresses, players gradually adapt to the match rhythm, and their physical and psychological condition may decline due to the cumulative effects of earlier matches, possibly resulting in fewer fouls compared to the season's outset. While previous studies have demonstrated differences in football players' running and technical performance in different air temperatures and stages (Chmura et al., 2021; Li et al., 2023; Özgünen et al., 2010), no study has examined the variations in the number of fouls under these two conditions.

Based on the previous review, we found that the referee's running performance, team quality difference, temperature, and league stages may influence the number of fouls. Therefore, the purpose of this study was to (1) examine the impact of referees' running performance during matches (running distance of the referee, decision distance of the referee) and the difference in teams' quality on the number of fouls; (2) explore whether there are significant differences in the number of fouls under various air temperature conditions and at different stages of the season. The findings of this research could academically advance our understanding of the factors that impact the number of fouls in soccer games. Particularly, we explore how referees' running performance, team quality difference, game stage, and air temperature influence the number of fouls in soccer matches. The findings of this study provide valuable practical implications for referees, thereby helping them predict the frequency of fouls, develop effective running strategies to reduce the number of fouls, and finally increase the smoothness of the game.

## Method

### Sample size calculation

Before data collection, we employed a sample size calculation using G^*^Power software (version 3.1.9.7) to ensure adequate statistical power. Based on the previous study on environmental factors influencing number of fouls (Yuan et al., 2024), we anticipated a small to moderate effect size (*f*^2^ = 0.15) in our study (Cohen, 2013). Following Hopkins et al. (2009) suggestion, we set the desired statistical power at 0.80 with an alpha level of 0.05. A linear bivariate correlation analysis was performed, which determined a minimum sample size of 270 matches. Our final sample of 480 games substantially exceeded this requirement, providing robust statistical reliability for the study.

### Match samples

Data were collected from 480 matches of the Chinese Super League (CSL) during the 2018 and 2019 seasons, when the VAR technology had been fully implemented (Han et al., 2020). The CSL is the top professional soccer league in China, consisting of 16 teams. It runs from March to November each year. The league employs a double round-robin system where each team plays 30 matches per season, totaling 480 matches. The scoring system awards 3 points for a win, 1 point for a draw, and 0 points for a loss. At the end of the season, the team with the highest points is crowned league champion (Gong et al., 2021). This study received approval from the Ethics Committee of Capital University of Physical Education and Sports.

### Inclusion and exclusion criteria

All 480 matches from the 2018–2019 CSL seasons were initially included. Matches were excluded if there was any missing data (e.g., the air temperature could not be found from the China National Environmental Monitoring Center). Because no missing data were detected, all 480 games were retained for analysis.

### Data collection

Referee running performance data were collected using the Amisco Pro^®^ semi-automated computer tracking system, which simultaneously tracked and recorded referees' physical performance, including total running distance, high-speed and low-speed running distances, and decision distances. This setup uses multiple fixed cameras to track all on-field participants, with specialized software analyzing the footage. The reliability and validity of the Amisco Pro^®^ system have been discussed in detail in prior studies (Zubillaga et al., 2008). We sourced data on the strength difference between teams and number of fouls from WhoScored (http://www.whoscored.com), which has been regarded as the most comprehensive soccer dataset, incorporating multiple national soccer leagues' information (Kessouri and Dachri, 2021). Concretely, the strength difference between teams was calculated by subtracting the seasonal point rankings of the two teams, a method commonly used in soccer technical and tactical analysis studies (Li et al., 2020; Zhou et al., 2019). While the number of fouls was calculated by adding the total fouls committed by the two teams. Temperature data were obtained from the China National Environmental Monitoring Center, which publishes real-time weather and air quality information. We calculated match air temperatures by averaging the air temperature from kickoff to final whistle. Following James et al. (2022) criteria, we categorized temperatures into four brackets: Cool: Up to 20.0°C, Warm: 20.1–25.0°C, Hot: 25.1–30.0°C, Very Hot: Above 30.0°C. The whole season was divided into three equal phases: Stage 1 (Rounds 1–10), Stage 2 (Rounds 11–20), and Stage 3 (Rounds 21–30).

### Data analysis

For statistical analysis, we first utilized the Shapiro-Wilk test to examine the normality of the data, then described the research sample using means and standard deviations (SD). As the running distance and decision distance of referees followed a normal distribution, while the quality difference between teams did not, we employed Pearson correlation analysis to examine the relationships between referee running distance and number of fouls, as well as between decision distance and foul count. Spearman correlation analysis was utilized to examine the relationship between strength difference between teams and number of fouls. Relationships were categorized as; trivial: 0.00–0.09; small: 0.10–0.29; moderate: 0.30–0.49; large: 0.50–0.69; very-large: 0.70–0.89; nearly perfect: 0.90–0.99; and perfect: 1.00 (Hopkins et al., 2009).

Linear regression was conducted to determine how referee running distance, decision distance, and team strength difference (independent variables) predicted the number of fouls committed by players (dependent variable). In addition, the difference in number of fouls across different temperature ranges and stages of the season was analyzed using one-way ANOVA with Bonferroni *post-hoc* tests. All statistical analyses were conducted using SPSS (version 23, SPSS Inc, USA), with the significance level set at *p* < 0.05.

## Results

### Descriptive statistics

The definition of variables are listed in Table 1. The descriptive statistics, correlation analysis, and regression model results for referees' running performance, strength difference between teams, and number of fouls were presented in Table 2.

**Table 1:** Definition of variables.

**Variables**	**Definition**
Number of fouls	The total number of fouls committed by two teams in a single match
Running distance of the referee (m)	The total distance covered by the main referee during active play in a single match, excluding movement when the ball is out of bounds or after the referee has blown the whistle to stop play
Decision distance of the referee (m)	The average distance between the referee and the spot of the foul when making the foul decision in a match
Strength difference between teams	The rank difference in the final of the season
Air temperature (°C)	The average the air temperature from kickoff to final whistle
Stage	1–10 rounds refer to stage 1, 11–20 rounds refer to stage 2, 21–30 refers stage 3

**Table 2:** Mean ± SD, correlation, and regression model of number of fouls, running distance of the referee, decision distance of the referee, and strength difference between teams.

**Variable**	**Mean ±SD**	**Relationship with number of fouls**	**Relationships with running distance of the referee**	**Relationships with decision distance of the referee**	**Relationships with strength difference between teams**	**Regression model (number of fouls)**
Number of fouls	29.25 ± 6.5	–				
Running distance of the referee	7,058.79 ± 774.32	−0.279^**^	–			b_0_ = 45.801 b_1_ = −0.002 SEE = 6.351
Decision distance of the referee	14.7 ± 1.44	0.024	−0.204^**^	–		
Strength difference between teams	5.67 ± 3.64	−0.011	0.117^*^	−0.083	–	

^*^p < 0.05; ^**^p < 0.01.

**Figure 1:**
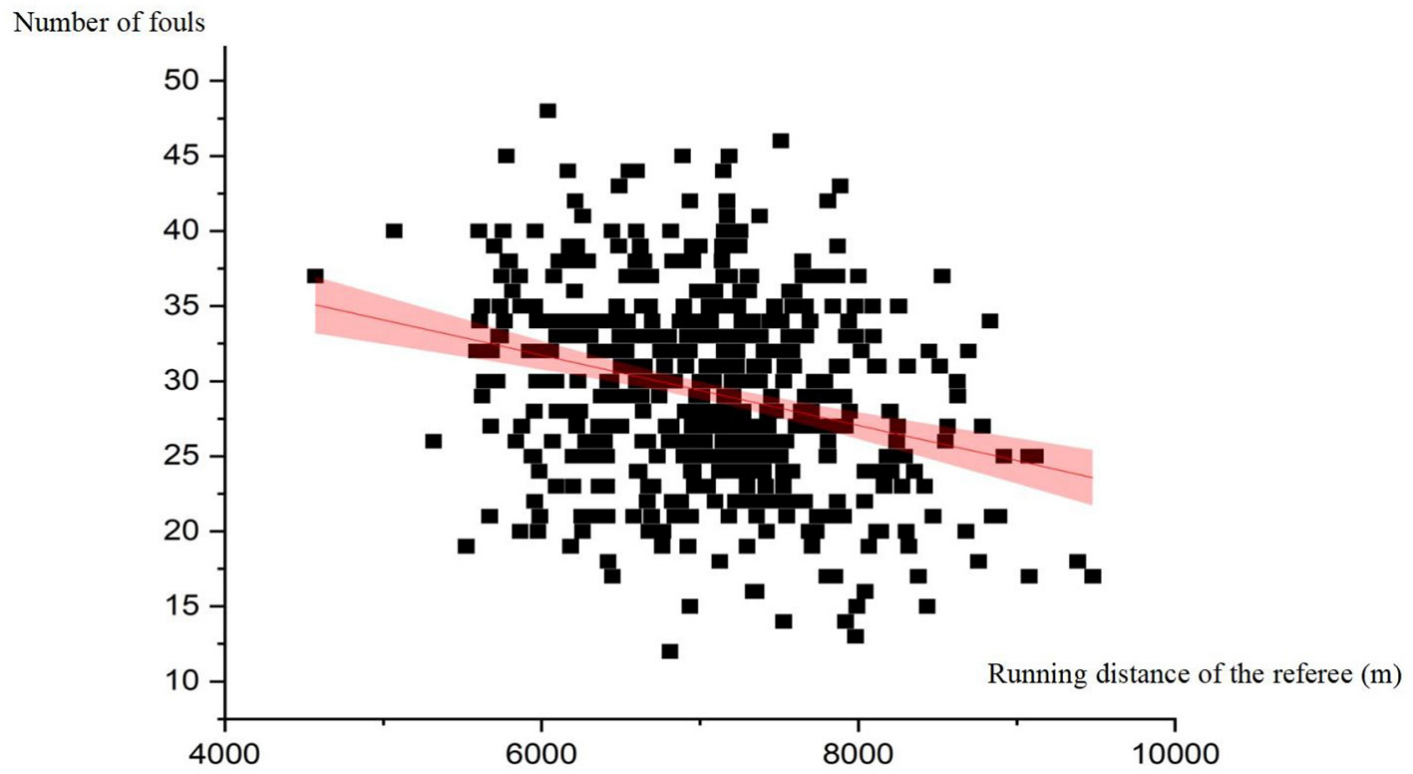
Panel of scatter plots demonstrating the relationship between running distance of the referee and number of fouls.

The correlation analysis revealed a significant negative relationship only between the running distance of the referee and the number of fouls (*r* = −0.279, *p* < 0.01). No significant associations were found between the decision distance of referees or the strength difference between teams and the number of fouls (*p* > 0.05). In addition, the linear regression analysis further supported the relationship between running distance of the referee and number of fouls. The regression equation is: Number of fouls = 45.801 – 0.002 ^*^ Referee running distance, with a standard error of estimate (SEE) of 6.351. Figure 1 illustrates the linear relationship between running distance of the referee and the number of fouls.

**Figure 2:**
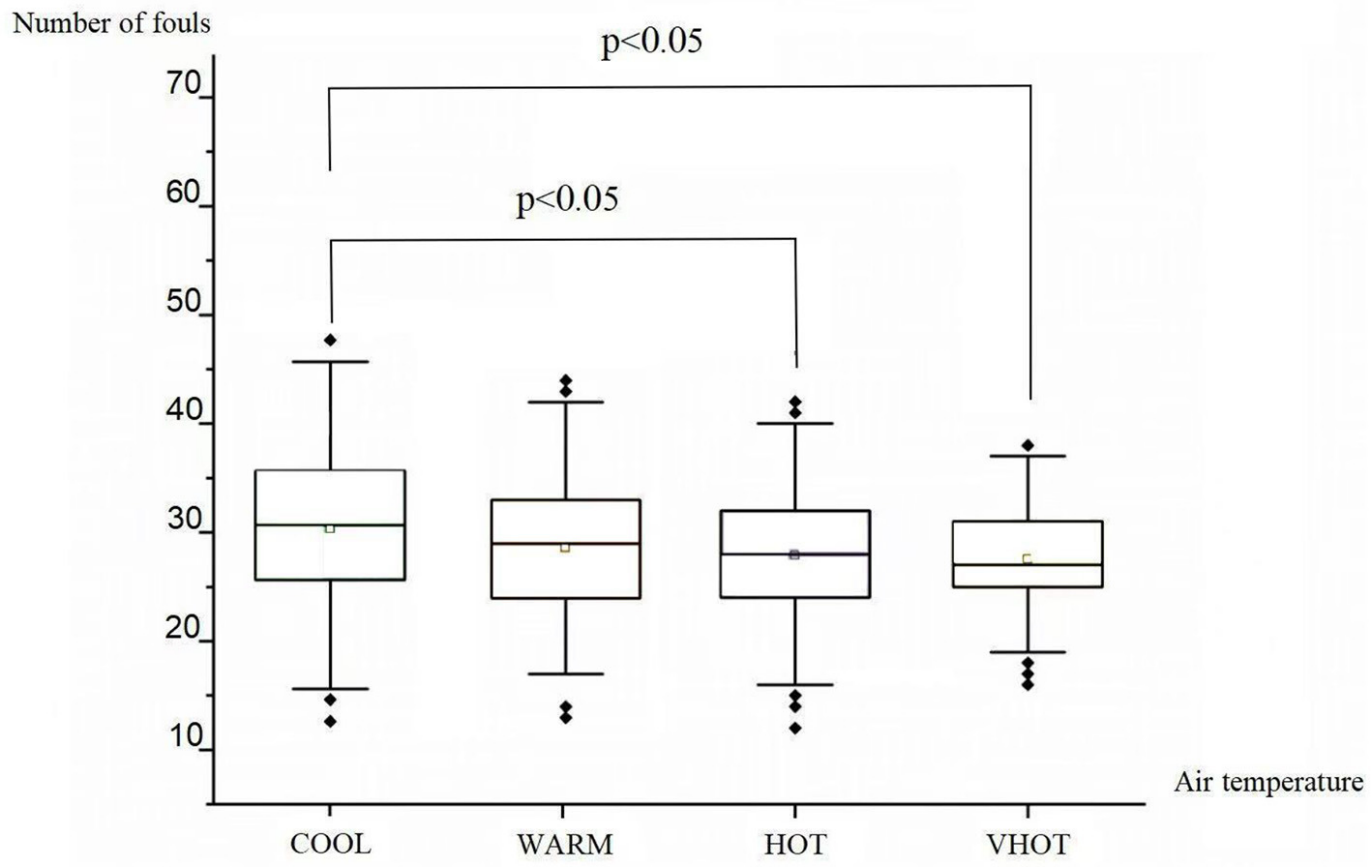
Comparison of number of fouls in different air temperature.

One-way ANOVA results demonstrated a significant difference in the number of fouls under different temperature conditions (*F* = 6.67, *p* < 0.001). Bonferroni *post-hoc* tests further revealed that matches played in COOL had significantly more fouls (30.67 ± 6.727) compared to those in HOT (27.86 ± 6.047, *p* < 0.05) and VHOT (27.55 ± 5.535, *p* < 0.05). No significant differences in number of fouls were found between WARM (29.63 ± 6.72) and HOT or VHOT (*p* > 0.05). Refer to Table 3 and Figure 2 for detailed information.

**Table 3:** Comparison of number of fouls in different air temperature.

**Air temperature (°C)**	***N***	**Number of fouls**	***F***	***P***	**Bonferroni *post-hoc* analyses**
COOL (air temperature ≤ 20.0°C)	179	30.67 ± 6.727	6.67	^**^	COOL < HOT^*^ COOL < VHOT^*^
WARM (20°C > air temperature ≤ 25.0°C)	105	29.63 ± 6.717				
HOT (25°C > air temperature ≤ 30.0°C)	132	27.86 ± 6.047				
VHOT (air temperature > 30.0°C)	64	27.55 ± 5.535				

^*^p < 0.05; ^**^p < 0.01.

**Figure 3:**
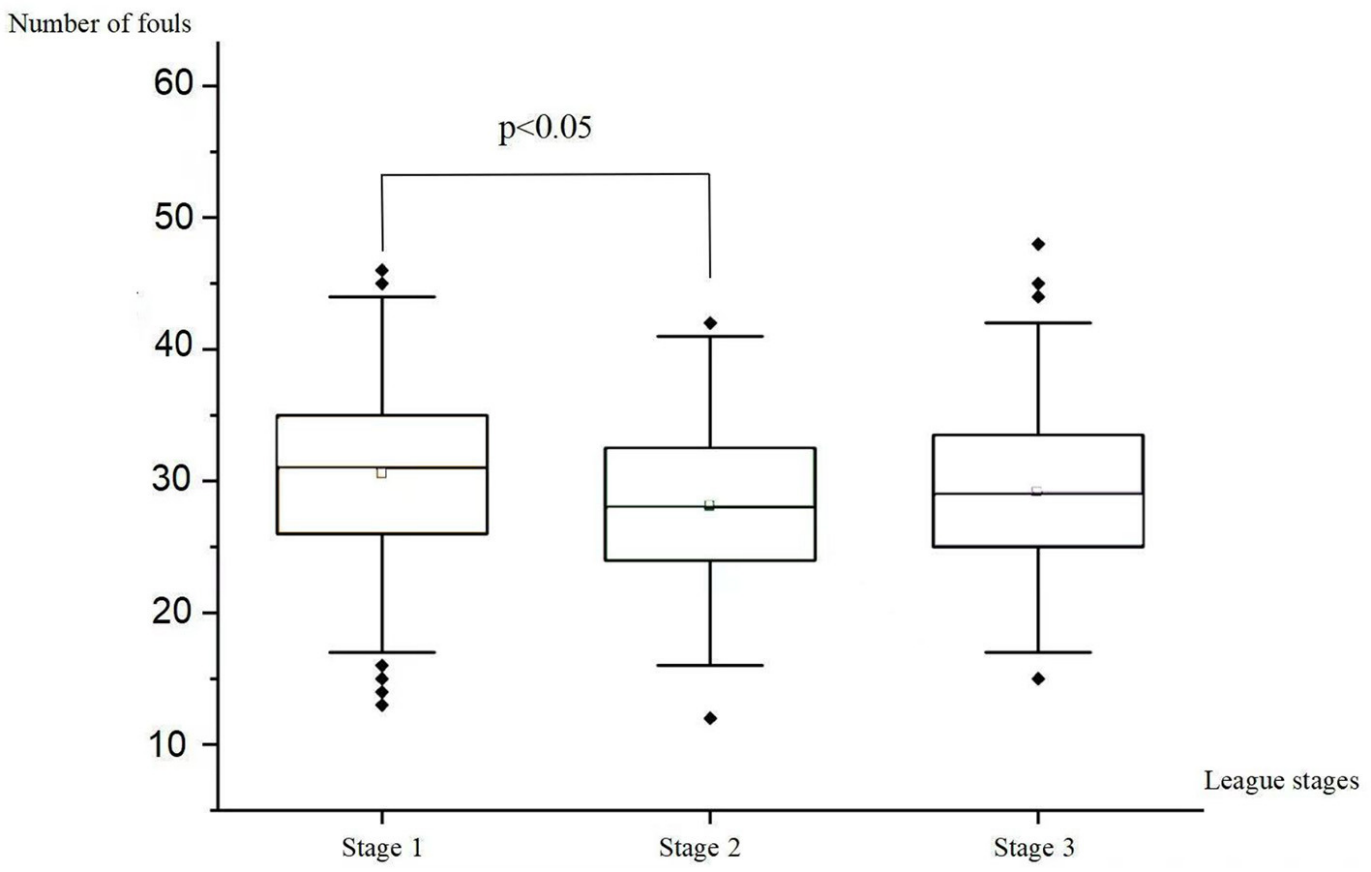
Comparison of number of fouls in different stage in a season.

A significant difference in number of fouls across different stages of the season was found (*F* = 5.810, *p* = 0.003). Bonferroni *post-hoc* tests further indicated Stage 1 saw notably more fouls per match (30.54 ± 6.92) than Stage 2 (28.10 ± 6.03, *p* = 0.002). No significant difference was uncovered between Stage 1 and Stage 3 (*p* > 0.05). Similarly, Stage 2 and Stage 3 showed a similar number of fouls (*p* > 0.05). Refer to Table 4 and Figure 3 for detailed information.

**Table 4:** Comparison of number of fouls in different stage in a season.

**Stage**	***N***	**Number of fouls**	***F***	***P***	**Bonferroni *post-hoc* analyses**
Stage 1 (round 1–10)	160	30.54 ± 6.921	5.81	^*^	COOL < HOT^*^ COOL < VHOT^*^
Stage 2 (round 11–20)	160	28.1 ± 6.026				
Stage 3 (round 21–30)	160	29.25 ± 6.508				

^*^p < 0.05.

## Discussion

This study examined how the referees' running performance, difference in team quality, temperature, and league stages affect the number of fouls in soccer matches. Our findings contribute academically to extending the understanding of foul occurrences in soccer. From a practical perspective, the results provide valuable information for referees to evaluate games and formulate strategies to reduce the number of fouls and improve game quality.

The current study uncovered a significant negative correlation between referees' running distance and the number of fouls (*r* = −0.279, *p* < 0.01). This finding aligns with our hypothesis and supports the strict physical requirements set by the referee administration department for referees (Chiu, 2022). The regression model further provided evidence for this relationship: Number of fouls = 45.801 – 0.002 ^*^ Referee running distance (SEE = 6.351). The results show that if referees run an additional 1 kilometer during the game, the number of fouls decreases by 2. Although the effect size is small, it highlights the potential impact of referee movement on the course of the game. This finding can be explained in several ways. Firstly, active running during the game provides referees with a better viewing angle, improving the accuracy of calls and convincing players (Mallo et al., 2010). In addition, active running creates more opportunities for referees to communicate with players (Simmons, 2006), thereby alleviating player dissatisfaction with the referee in a timely manner and defusing potential conflicts between players, which in turn helps to reduce subsequent retaliatory fouls arising from anger.

Surprisingly, no significant correlation was found between the number of fouls and either the decision distance of the referee or the strength difference between teams, which is not aligned with our initial hypothesis. The lack of correlation between decision distance and fouls could stem from the non-linear relationship between decision distance and accuracy. While Mallo et al. (2012) and Hossner et al. (2019) suggested optimal decision distances of 10–15 m, where referees make fewer errors, De Oliveira et al. (2011) found no significant relationship between the decision distance of the referee and foul accuracy when using continuous numeric variables to represent the running distance of referees. Similarly, Riiser et al. (2017) also reported that the decision accuracy is not related to referees' position in relation to the infringements. Therefore, we may assume that despite referees being in close proximity, players may still commit fouls as a form of retaliation, if the referee's previous decision was perceived as incorrect (Mark et al., 1983).

The absence of a significant relationship between the strength difference between teams could be explained by the fact that season-end rankings may not accurately represent the importance of a game at a specific time. For example, a team facing imminent relegation may play aggressively against other teams (e.g., with frequent body contacts and fouls) in the first stage, as they are eager to earn points. In contrast, the same team might play more relaxedly and with less intensity (e.g., fewer body contacts and fouls) toward other teams when the team is doomed to be relegated in the last stage, even when the teams' strength difference remains close at the end of the season.

Our study also found a significant difference in the number of fouls across varying temperature conditions (*F* = 6.669, *p* < 0.001). In particular, the number of fouls was more prevalent in cooler temperatures compared to hotter and warmer temperatures. Previous research has shown that temperature plays a role in influencing athletes' physical performance (Guy et al., 2015). In hot environments, athletes experience heightened metabolic rates, which can reduce their explosiveness and endurance, potentially impacting athleticism and aggressiveness (Harper et al., 2019; Stewart et al., 2019). This may reflect a mechanism by which the number of fouls is indirectly influenced by air temperature through athletes' performance conditions. When the temperature is cool, players' mental and athletic performance is optimal (Kang et al., 2024), leading to increased body contacts and consequently a higher number of fouls. Conversely, when the air temperature is hot or very hot, athletes' mental performance decreases and their physiological functions decline (Kang et al., 2024), resulting in a reduction in the number of fouls.

Furthermore, our findings supported the hypothesis that the number of fouls in Stage 1 (1–10 rounds) was significantly higher than in Stage 2 (11–20 rounds; *p* = 0.002). This result can be attributed to fluctuations in physical and psychological conditions. Carling et al. (2015) noted that football players exhibit decreased physical and technical performance toward the end of the season. In contrast, players are typically well-prepared in terms of fitness and mindset at the season's commencement, potentially leading to increased body contact and confrontations, resulting in more fouls. After experiencing 10 rounds, players' physical fitness drops, and motivation wanes, resulting in fewer fouls.

Based on our findings, we recommend that referee management departments continue to stress the importance of referees' fitness and advocate the role of referees' running performance in reducing the occurrence of fouls. Referees' running distance can be acknowledged as one of the criteria for promotion and rewards. In addition, referees are encouraged to be carefully prepared both mentally and physically when officiating matches under 20°C and at the beginning stage of the season.

## Limitations and future directions

Although this study significantly contributes to foul studies in football literature, several limitations remain. First, the data used was exclusive from the CSL, therefore limiting the generalizability of the findings. Moreover, different referees may have varying standards for foul commitment, which may undermine the validity of the data. Therefore, future studies may include soccer games outside of China (e.g., European soccer leagues and World Cup). Third, the air temperature was categorized according to James et al. (2022) criteria, which was conducted in Malaysia where the athletic environment is different from China (e.g., humidity and altitude), consequently, future studies may reference environmental criteria based on local studies. Fourth, we evidenced running distance negatively associated with number of fouls, however the effect size is small. In addition, decision distance and game stage were found not to relate to the number of fouls. Consequently, more factors, especially contextual factors, are encouraged to be included in future studies. Building on Goumas's (2012) finding that crowd density could result in referees' bias, future studies may incorporate stadium attendance and running track to explore environmental effects on the number of fouls in soccer games.

## Conclusion

This study revealed that referees' running distance in games negatively relates to the number of fouls. Additionally, more fouls occurred when games were played in cool environment situation and in the early stage of the season. No significant relationship was found between the number of fouls and either the decision distance of the referee or the strength difference between teams. The finding not only pioneers research on the number of fouls from the perspective of referees' running performance, weather conditions, and stage, but also extends the understanding of the patterns of foul occurrences. The findings of the study also offer practical implications for referees to predict the frequency of fouls, thereby enabling better game preparation based on weather conditions and the stage of the game. The negative relationship between running distance and number of fouls provides insights into running strategies to reduce foul occurrences.

## Data Availability

The raw data supporting the conclusions of this article will be made available by the authors, without undue reservation.
